# Histology and Ultrastructure of the Esophagus in European Beaver (*Castor fiber*) Displays Features Adapted to Seasonal Changes in Diet

**DOI:** 10.3390/ani13040635

**Published:** 2023-02-11

**Authors:** Kamila Martyniuk, Natalia Ziółkowska, Maria Hanuszewska-Dominiak, Natalia Szyryńska, Bogdan Lewczuk

**Affiliations:** Department of Histology and Embryology, Faculty of Veterinary Medicine, University of Warmia and Mazury in Olsztyn, Oczapowskiego 13, 10-719 Olsztyn, Poland

**Keywords:** epithelium, keratinization, histology, ultrastructure, PCNA, rodent, wildlife

## Abstract

**Simple Summary:**

The European beaver is the biggest rodent in Eurasia and its diet is closely related to the season of the year. The gastrointestinal tract adapts its structure and physiology to ensure resistance to mechanical factors and proper digestion. Among mammals, the esophagus structure is highly variable. We aimed to perform morphological analysis of the esophagus in the European beaver, with a special attention to differences in the thickness of the epithelium in organs collected in spring, summer, and winter. Our results reveal that the mucosa is lined with stratified squamous keratinized epithelium with a structure similar to that of the skin epidermis. The process of epithelial keratinization occurs in both adult and fetal animals, suggesting that it is genetically programmed. The keratinized layer is much thicker in esophagi collected in winter than in spring and summer, while the living cell layer thickness remains unchanged regardless of the season. Moreover, immunohistochemical staining shows increased proliferation of epithelial cells in winter compared to that in spring and summer. Ultrastructural studies reveal the presence of multiple lamellar and non-lamellar bodies in granular cells, whose morphology and location gradually change while reaching the upper epithelial layers. The morphology of the esophagus in the European beaver displays features adapted to seasonal changes in diet. Future studies could employ biochemical characterization of keratinization process in the epithelium and composition of keratinized layer.

**Abstract:**

The European beaver is a herbivorous rodent whose diet changes seasonally, and in winter consists of large quantities of woody plants. It is distinguished among other mammals by a unique organization of the stomach that comprises the cardiogastric gland and by the unusual process of mucus formation in the gastric mucosa. The aim of study was to (i) characterize the structure of the beaver esophagus with particular attention to the mucosal epithelium; (ii) compare the histological structure of the esophagi collected in spring, summer, and winter; (iii) provide preliminary data on the structure of the esophagus in beaver fetuses. The study was conducted on esophagi of 18 adult beavers captured in Poland in April, August, and December, and on 3 fetal organs. The results obtained in adults show that the mucosa is lined with thick stratified squamous keratinized epithelium with a structure similar to that of the skin epidermis. Ultrastructural studies reveal the presence of multiple lamellar and non-lamellar bodies in granular cells, whose morphology and location gradually change while reaching the upper epithelial layers. The muscularis mucosa comprises a layer of longitudinally oriented bundles of smooth muscle cells. Both mucosa and submucosa do not comprise any glands. The thick muscularis externa consists mainly of internal circular and external longitudinal layers of striated muscle fibers. The keratinized layer of mucosa epithelium was 2-3-fold thicker in esophagi collected in winter than in those collected in spring and summer, while the epithelial cell layer thickness remained unchanged regardless of the season. Immunolabeling for proliferating cell nuclear antigen shows a higher index of epithelium proliferation in esophagi collected in winter than in spring and summer. No seasonal differences were noted in other layers of the esophagus. Fetal organs have epithelium covered with a keratinized layer, thinner than in adults, and the muscularis externa comprises both striated and smooth muscle cells.

## 1. Introduction

The structure of a mammalian esophagus shows prominent interspecies differences, which concern the keratinization of mucous epithelium, the arrangement of muscularis mucosa, the presence of mucus-secreting glands, and the organization of muscularis externa [1,2,3,4,5,6]. The differences are closely related to physicochemical properties of food and the digestive physiology, however, several aspects of these relationships remain unknown.

The European beaver (*Castor fiber*) is the largest wild rodent species in Eurasia, belonging to the suborder *Castorimorpha* and the family *Castoridae* (together with the second member of this family, *Castor canadensis*). As a herbivorous animal, it feeds mainly on plants, and the type of food it consumes is closely related to the season of the year. In summer, beavers eat leaves and young sprouts, while in winter, their basic food consists of large quantities of woody plants [7,8,9]. Recent research conducted on the European beaver revealed uncommon features of its stomach including the presence of the cardiogastric gland, which has been described in less than 20 mammalian species, and the occurrence of distinctive mucus layer covering the mucosa [10]. This gland is located close to the entrance of the esophagus to the stomach and comprises mainly parietal cells and chief cells [10]. The formation of mucus covering the stomach mucosa is also a species-specific process and includes the secretory granule accumulation in cytoplasm of pit cells, the granule aggregation inside cells, and the incorporation of degenerating cells into the mucus [10]. The very thick layer of this mucous is obviously a highly effective, protective barrier against hard food particles and gastric juice [10,11]. The analysis of unique features of the stomach leads to the question if the beaver’s esophagus has some special protective barriers.

Taking into account the distinctness of the European beaver’s lifestyle and several adaptive structural features of its gastrointestinal tract due to the large uptake of woody parts [10,12], the goal of the present study was to (i) characterize the structure of the beaver esophagus with particular attention to histology and ultrastructure of the mucosal epithelium; (ii) compare the histological structure of the esophagi collected in spring, summer, and winter; (iii) provide preliminary data on the structure of the esophagus in beaver fetuses.

## 2. Materials and Methods

### 2.1. Ethics and Legal Statement

The study was performed in strict accordance with the Polish and EU laws of animal welfare and protection. The animal capture and euthanasia were approved by the Regional Directorate for Environmental Protection in Olsztyn, a government institution responsible for the management of wildlife in the Warmia and Mazury Voivodship of Poland. Animal captures were allowed due to beaver overpopulation in this area and were conducted by a specialized team from the Polish Hunting Association. The experimental protocol including animal euthanasia was approved by the Local Ethical Commission for Experiments on Animals at the University of Warmia and Mazury in Olsztyn (Permit Numbers 22/2007, 33/2009) and the III Local Ethical Commission for Experiments on Animals at Warsaw University of Life Sciences (Permit Number 11/2010). All possible efforts were made to minimize animal suffering.

### 2.2. Animals

The study was conducted on 18 adult European beavers captured in their natural habitat in northeastern Poland in April (n = 6), August (n = 6), and December (n = 6). Beavers were euthanized by exsanguination under deep anesthesia induced by intramuscular administration of 60 mg of xylazine (Sedazin, Biowet Puławy, Puławy, Poland) and 300 mg of ketamine (VetaKetam, VetAgro, Lublin, Poland). Esophagi were removed immediately after the heart stopped and used for histological, immunohistochemical, and ultrastructural studies. During experiment, it was discovered that two females were pregnant, thus, three fetuses in the last prenatal development stage were collected for histological and immunohistochemical studies.

### 2.3. Histology and Immunohistochemistry

Tissue samples were taken from the anterior, middle, and posterior parts of esophagi, then fixed in 4% paraformaldehyde in 0.1 M phosphate buffer (pH 7.4) for 48 h, dehydrated in ethanol (TP 1020, Leica, Wetzlar, Germany), and embedded in paraffin (EG1150, Leica, Wetzlar, Germany). The 4 µm thick sections were prepared (RM2125, Leica, Wetzlar, Germany) and stained with hematoxylin and eosin (HE) using an automated multi-stainer ST 5020 with cover-slipper CV5030 (Leica, Wetzlar, Germany). The slides were digitalized using Pannoramic 250 Flash scanner (3DHistech, Budapest, Hungary).

Immunohistochemistry was performed on paraffin sections (after wet-heat-induced epitope retrieval in Tris-EDTA buffer, pH 9) using primary antibodies against proliferating cell nuclear antigen (PCNA, diluted 1:200, clone PC10, M0879, Dako Agilent Technologies, Santa Clara, CA, USA), human smooth muscle actin (SMA, diluted 1:50, clone 1A4, Dako, Glostrup, Denmark), and human desmin (diluted 1:50, clone D33, Dako, Glostrup, Denmark). The reaction products were visualized using the immunoperoxidase method with 3,3-diaminobenzidine (DAB) or 3-amino-9-ethylcarbazole (AEC) as the substrates. The specimens were counterstained with Mayer’s hematoxylin and cover slipped using Mounting Glycergel Medium (Dako, Glostrup, Denmark) or Canadian balsam. For negative controls, the primary antibody was replaced with mouse IgG1 or IgG2a (Dako, Glostrup, Denmark) at the appropriate dilution.

### 2.4. Morphometric Evaluation

Morphometry was performed on HE-stained cross-sections from the anterior (3rd cm from the pharynx), middle, and posterior (3rd cm from the stomach) parts of esophagi of adult animals collected in April, August, and December. From each part of the organ, ten sections (separated each other by 150 µm) were used in the measurements of the thicknesses of whole mucosa, the keratinized layer, the epithelial cell layer, the muscularis mucosa, the submucosa, the muscularis externa, and the whole esophagus wall. Linear measurements were repeated 10 times per section for each parameter. The analyses were performed using the Panoramic Viewer 1.15 software program (3D-Histech, Budapest, Hungary). 

### 2.5. Degree of Cellular Proliferation

PCNA index was determined to evaluate the degree of cellular proliferation of epithelial cells in the middle part of esophagi collected in April, August, and December from adult beavers. For comparison, the fetal esophagi were also included in this analysis. PCNA index was calculated as the percentage of PCNA-positive nuclei in the total number of nuclei [(PCNA-positive nuclei count/total nuclei count) × 100%]. The nuclei were manually counted in four rectangle 100 µm × 400 µm located over the epithelial cell layer in each section. Five sections per esophagus were analyzed. 

### 2.6. Ultrastructural Studies

The samples of mucous membranes were collected for electron microscopy within 3 min after the heart stopped. Tissue pieces were fixed (2 h, 4 °C) in a mixture of 1% paraformaldehyde and 2.5% glutaraldehyde in 0.2 M phosphate buffer (pH 7.4). Samples were then washed, post-fixed in 2% osmium tetroxide (two hours at room temperature), and embedded in Epon 812. Semi-thin sections were cut from each block of tissue, stained with toluidine blue, and examined to identify regions for electron microscopy. Ultrathin sections were contrasted with uranyl acetate and lead citrate and then examined using a Tecnai 12 Spirit G2 BioTwin transmission electron microscope (FEI, Hillsboro, OR, USA) equipped with two digital cameras: Veleta (Olympus, Tokyo, Japan) and Eage 4k (FEI, Hillsboro, OR, USA).

### 2.7. Statistical Analysis 

The data were analyzed by two-way analysis of variance with the season and the region of the organ as factors. The Duncan test was used a post-hoc procedure. Statistical significance was set at *p* < 0.05. The analyses were performed using Statistica 13 software (TIBCO Software, Inc., Palo Alto, CA, USA).

## 3. Results

### 3.1. Histology

**Figure 1:**
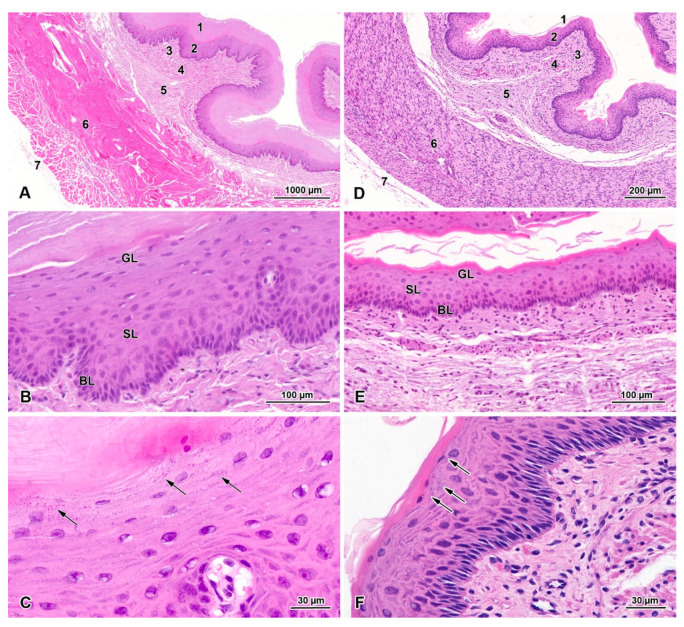
Histological structure of the adult and fetal beaver esophagus. Hematoxylin and eosin staining (HE). (**A**) Histological structure of the esophagus in adult European beaver: 1—thick keratinized layer; 2—epithelial cell layer; 3—lamina propria; 4—muscularis mucosa; 5—submucosa; 6—muscularis externa; 7—adventitia. (**B**) Histological structure of epithelial cell layer in adult beaver esophagus: BL—basal layer; SL—spinous layer; GL—granular layer. (**C**) Magnification of granular layer. Note the presence of basophilic inclusions in the cytoplasm of the granular cell (arrows). (**D**) Histological structure of the esophagus in fetal European beaver: 1—thin keratinized layer; 2—epithelial cell layer; 3—lamina propria; 4—muscularis mucosa; 5—submucosa; 6—muscularis externa; 7—adventitia. (**E**) Histological structure of epithelial cell layer in fetal beaver esophagus: BL—basal layer; SL—spinous layer; GL—granular layer. (**F**) Magnification of granular layer. Note the presence of basophilic inclusions in the cytoplasm of the granular cell (arrows).

The mucosa and submucosa form loosely arranged longitudinal folds (Figure 1A,D). In adult beavers, the esophageal mucosa is lined with stratified squamous epithelium composed of an epithelial cell layer covered by a very thick keratinized layer. In the epithelial cell layer, three sublayers comprising morphologically distinct epithelial cells could be distinguished (Figure 1B). The first layer (basal layer) of cells lying on the basement membrane is composed of vertically arranged columnar cells containing an elongated nucleus. The next layer (spinous layer) is composed of a few sheets of cells of various shapes, equipped with a round nucleus containing a plain nucleolus. The last sublayer (granular layer) is composed of 3–5 sheets of cells filled with multiple basophilic granules (Figure 1C). The keratinized layer consists of flattened cells usually without a nucleus (Figure 1B). The flattened cells detach from the surface of the keratinized layer, presenting signs of desquamation (Figure 1A).

The lamina propria is formed by loose connective tissue and contained numerous blood vessels (Figure 1A). The muscularis mucosa comprises a discontinuous layer of longitudinally oriented bundles of smooth muscle cells. The number of these bundles increases towards the stomach. The submucosa has typical structure. We do not observe any kind of glands in submucosa. The thick muscularis externa consists mainly of internal circular and external longitudinal layers of striated muscle fibers. However, a few flat or oval bundles of longitudinally oriented striated muscle fibers are observed on the internal surface of the circular layer. The myenteric plexus, located between the longitudinal and circular layers, is well-developed. The tunica adventitia is composed of loose connective tissue with blood vessels surrounding the esophagus. The tunica serosa is present behind the esophageal hiatus.

**Figure 2:**
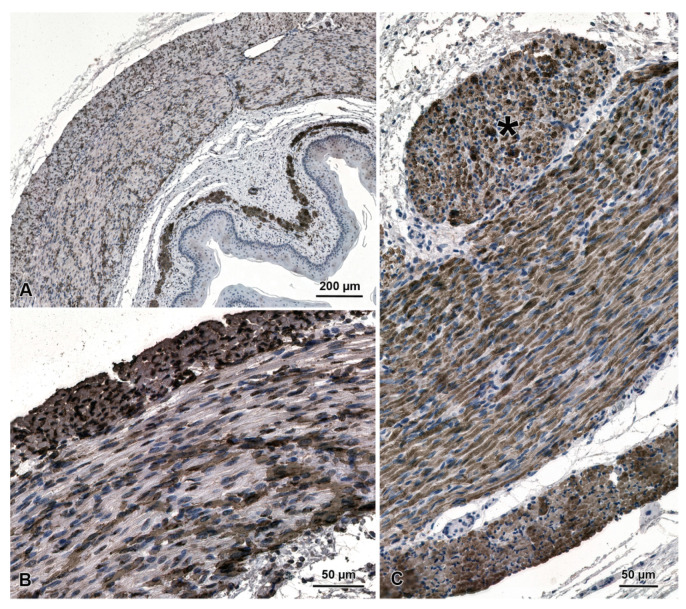
Distribution of smooth muscle and striated muscle cells in the esophagus of beaver fetuses demonstrated by immunohistochemical staining with antibodies anti-SMA (**A**,**B**) and anti-desmin (**C**). SMA-positive smooth muscle cells are visible both in the muscularis mucosa and the muscularis externa (**A**). Higher magnification of muscularis externa shows both types of muscle cells (**B**). Note an oval bundle of longitudinally oriented muscle cells on the internal surface of the circular layer (star in **C**).

The general structure of the fetal esophagus is similar to that observed in adult beavers (Figure 1D). The epithelial cell layer is much thinner than in adult animals and consists of one sheet of basal cells, 2–3 sheets of spinous cells, and 1–2 sheets of granular cells (Figure 1E,F). The stratified epithelium is covered with a layer of keratinized tissue, which is much thinner than in adult animals. In fetal esophagi, the muscularis externa comprises both striated muscle fibers and smooth muscle cells, which is clearly demonstrated by immunochemistry with antibodies against smooth muscle actin and desmin (Figure 2).

### 3.2. Morphometry

**Figure 3:**
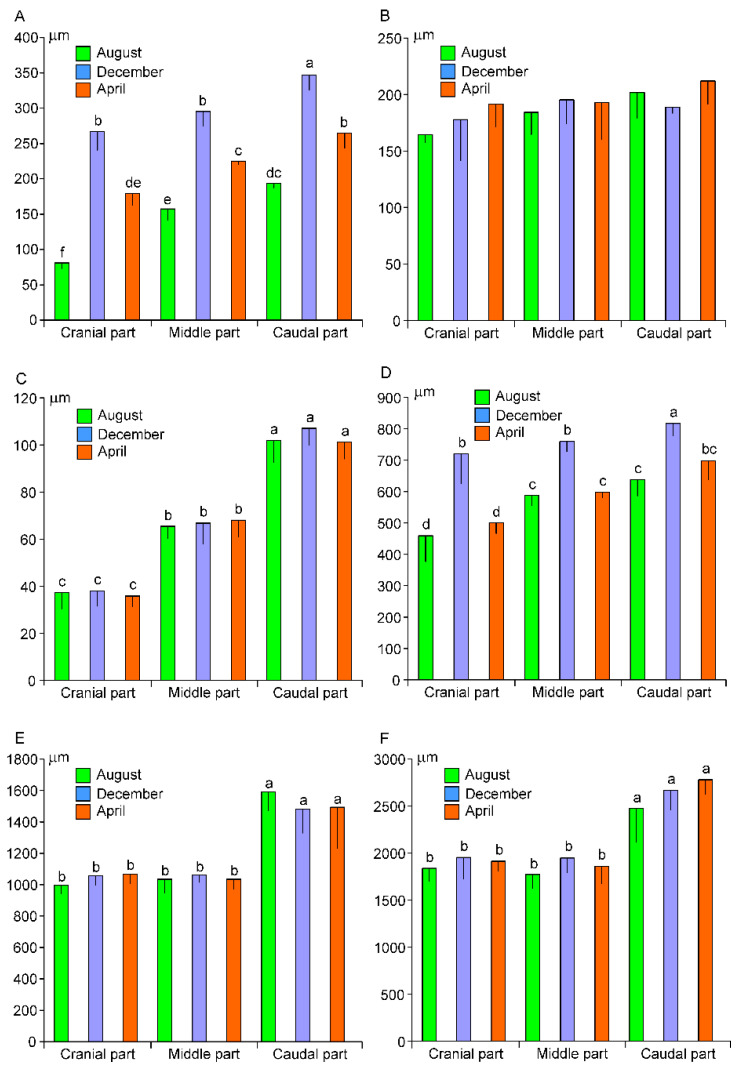
The thickness (means ± SD) of the keratinized layer (**A**), the epithelial cell layer (**B**), the muscularis mucosa (**C**), the mucosa (**D**), the muscularis externa (**E**), and the whole wall (**F**) in the esophagi of adult European beavers collected in August, December, and April. The same letters indicate means that are not significantly different.

Morphometric analysis shows significant differences in thickness of the keratinized layer depending on the part of the esophagus and the season (Figure 3A). The thickness of this layer increases from the cranial to the caudal part of the organ, irrespective of the time of year. Seasonal differences are the most prominent in the cranial part of the esophagus, where the thickness of keratinized layer is approximately 3-fold higher in December than in August and 1.5-fold higher in December than in April. Similar, but slightly lower, seasonal variations are found in the middle and cranial part of the esophagus. In contrast to the keratinized layer, the epithelial cell layer shows no significant differences of its thickness between parts of organ and seasons (Figure 3B). The thickness of muscularis mucosa increases significantly towards the stomach (Figure 3C). The seasonal and regional variations are also found in the measurement of the whole mucosa (Figure 3D). The thickness of muscularis externa is significantly higher in the caudal part than in cranial and middle parts of the esophagus (Figure 3E). Similar differences are found in the thickness of the whole esophagus wall (Figure 3F). 

### 3.3. PCNA Immunohistochemistry

**Figure 4:**
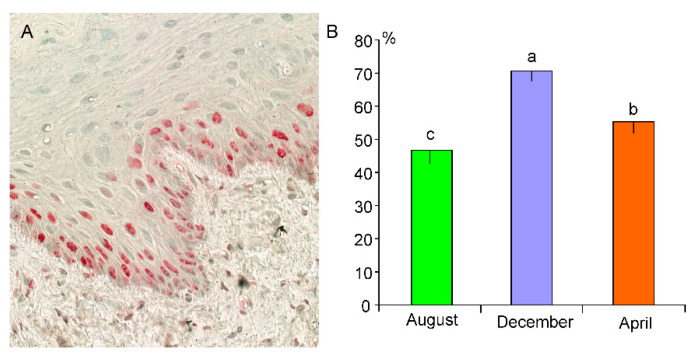
(**A**) PCNA-positive cells in the basal and spinous layers of the epithelium of adult beaver esophagi collected in April. (**B**) Percentage (means ± SD) of PCNA positive cells in the epithelial cell layers in esophagi of collected in August, December, and April. Different letters indicate significant differences between means.

Immunocytochemistry for PCNA shows a positive reaction in cells of the basal and spinous layers of the epithelium in both fetuses and adults esophagi (Figure 4A). The percentage of PCNA-positive cells is significantly higher in organs collected in December than in April and August, and in April than in August (Figure 4B). The highest PCNA index (92.1%) is found in the esophagi of fetuses.

### 3.4. Ultrastructure of Stratified Squamous Epithelium

**Figure 5:**
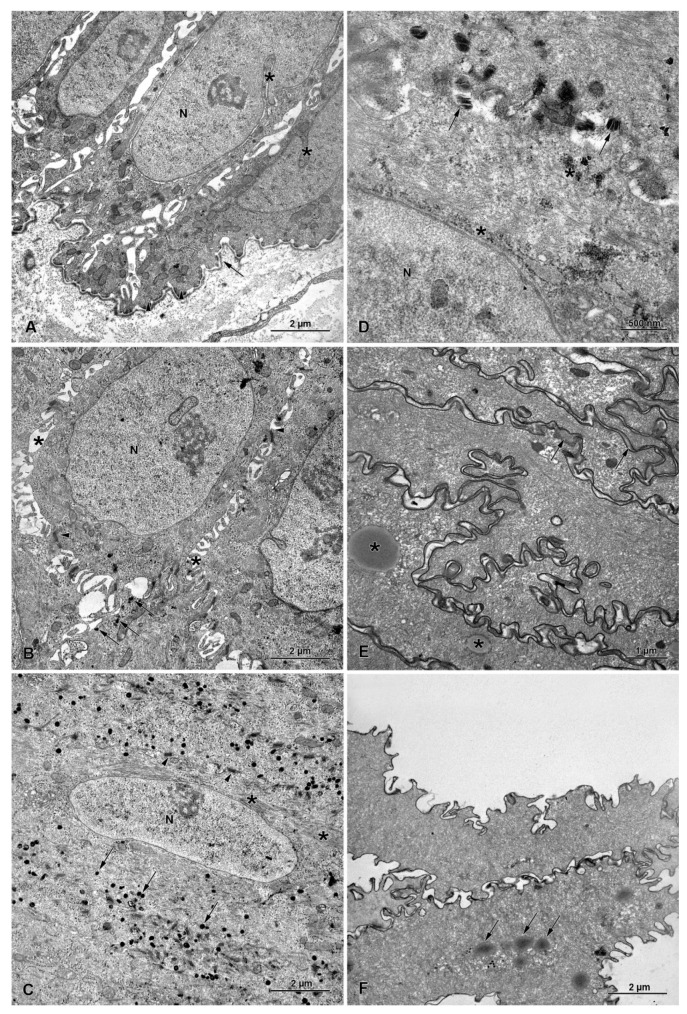
Ultrastructure of stratified squamous epithelium of the adult beaver esophagus. (**A**) Basal cells containing large nucleus (N) rich in euchromatin and nuclear envelope with deep invaginations (stars). Note that the attachment of these cells to the basement membrane (arrow) occurs via hemidesmosomes (arrowhead). (**B**) Spinous cells reveal multiple cytoplasmic processes (stars) and desmosomes (arrowhead). Note the presence of single, electron-dense bodies in the cytoplasm near the cell membrane (arrows). (**C**) Granular cells located near the spinous layer are filled with bundles of intermediate filaments (star) located around the nucleus (N). Numerous electron-dense bodies are present in cytoplasm, near the cell membrane (arrows). Note that granular cells are tightly connected to each other via desmosomes (arrowhead). (**D**) Granular cells located near the keratinized layer. Note that lamellar bodies spread out from granular cells (arrows) and occur in the extracellular space. Irregular areas of granular bodies are located in the cytoplasm (stars). Letter N shows nucleus (**E**) Keratinized layer contains lipid droplets (star), intermediate filaments, and the remains of granular and lamellar bodies. Note lamellar deposits in the intracellular space along interdigitated cell membrane (arrows). (**F**) Keratinized layer with cell desquamation visible as detaching from the surface. Note the presence of lipid droplets (arrow).

The basal layer consists of one layer of columnar cells that has an oval, euchromatin-rich nucleus with deep nuclear envelope invaginations and cytoplasm with rough endoplasmic reticulum, free ribosomes, and mitochondria (Figure 5A). Mitotic figures are occasionally observed in this layer. Basal cells are connected to the basal lamina of a typical structure by hemidesmosomes (Figure 5A).

The spinous layer consists of 4–6 layers of cells with dilated cisterns of rough endoplasmic reticulum filled with electron-dense content. The most prominent feature of this layer is the presence of numerous cell processes and desmosomes (Figure 5B). The bundles of intermediate filaments are present in the cytoplasm of spinous cells. Non-lamellar electron-dense bodies are occasionally observed in the cytoplasm of these cells (Figure 5B).

**Figure 6:**
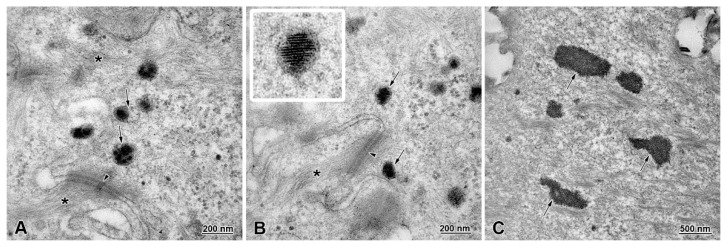
Ultrastructure of lamellar and non-lamellar bodies in the stratified squamous epithelium of the adult beaver esophagus. (**A**) Non-lamellar bodies present in granular cells are covered with membrane and have granular, electron-dense content and lucent clefts (arrows). Note long bundles of intermediate filaments (star) and desmosomes (arrowhead). (**B**) Lamellar bodies present in granular cells are round and ellipsoidal in shape (arrows). Insert shows electron-dense discs inside these bodies. Note bundles of intermediate filaments (star) and desmosomes (arrowhead). (**C**) Non-lamellar bodies present in keratinized layer consist of small electron-dense granules that form irregular shapes and are not covered with membranes (arrows).

The granular layer is composed of 2–3 layers of flattened cells, connected to each other by desmosomes (Figure 5C). The prominent feature of the granular layer is the presence of numerous electron-dense bodies and accumulation of intermediate filaments (Figure 5C). These bodies are mostly round in shape and located close to the cell membrane. Two types of bodies can be distinguished: (1) non-lamellar bodies with electron-dense granular content and electron-lucent clefts (Figure 6A); (2) lamellar bodies with electron-dense discs arranged horizontally and/or perpendicularly to the long axis of those bodies (Figure 6B). In the superficial region of the granular layer, the localization and morphology of these bodies changes (Figure 5D). Lamellar bodies are present very close to the cell membrane and in the extracellular space. In the cytoplasm, we could distinguish areas of small, electron-dense bodies that form irregular clusters (Figure 5D).

The keratinized layer consists of flattened cells without nuclei, filled with intermediate filaments. The ultrastructure of this layer shows the presence of the remains of non-lamellar bodies and multiple droplets of lipids (Figure 5E,F and Figure 6C). The cell membranes of keratinized cells are interdigitated. Lamellar structures and electron-dense material are present between these cells in the intracellular space (Figure 5E,F).

## 4. Discussion

The present study provides a description of morphological features of the esophagus of the European beaver at light and electron microscopy levels, paying a special attention to the differences between organs collected in spring, summer and winter. It also provides some data on the esophagus structure in fetuses. 

The obtained data show a typical histological structure with well-separated layers of the European beaver esophagus, both in adults and fetuses. The epithelium covering mucosa is characterized by heavy keratinization. The keratinized layer is very thick and the epithelial cell layer comprises the prominent granular layer with numerous basophilic granules. It should be noted that the histological organization of the esophageal epithelium is similar to the epidermis covering the skin [11]. The absence of nuclei in the cornified layer and the presence of a granular layer allow us to establish that it is an orthokeratinization and not a parakeratinization [13,14]. Keratinization is typical for the esophageal epithelia exposed to mechanical irritation by food compounds and is especially intensive in species with an abrasive diet, for example in rodents such as mice [15], rats [16,17], capybaras [18], guinea pigs [19], Syrian hamsters [20], grasscutters [21], and agoutis [22]; in domestic ruminants, such as cows [23], goats [24,25,26,27,28], and sheep [23,28]; in wild animals such as European roe deer [27], and in camels [1]. In contrast, a lack of keratinized layer in the esophageal epithelium has been described in mammals whose diet is based on a soft type of food, that is, humans [12], cats and dogs [12], buffalo calves [29], and rabbits [30]. In these animals, the superficial cells of the epithelium possess nuclei and only occasionally contain a few keratohyalin granules [31].

In the present study, morphometric analysis of the epithelial thickness in adult beavers reveals that the keratinized layer is 2–3-fold thicker in esophagi collected in winter than in those collected in summer. In spring, the keratinized layer thickness is between the values found in August and December. Such a difference is likely due to availability of different types of food in these seasons and confirms the adaptation of the epithelium to its protective function. In spring and summer, beavers eat green plant parts, and in winter, their diet is based on a large quantity of woody plants [7,8,9], which are much more mechanically irritating to the digestive tract. Interestingly, we also show that the keratinized layer is already present in the fetal esophagus, which suggests that the process of keratinization is genetically programmed. In contrast, in mice, the squamous epithelium becomes keratinized at least 8 days after birth [32,33].

Although a substantial difference in the thickness of the keratinized layer between adult esophagi collected in spring, summer, and winter is detected, no differences in the thickness of the epithelial cell layer between esophagi collected in those seasons are recorded. This observation leads to the assumption that in winter, due to increased epithelial keratinization and desquamation, the process of epithelial cell proliferation is more intense than in other seasons, ensuring the unaffected thickness of the epithelial cell layer. This hypothesis is strongly supported by PCNA immunolabeling, which shows a higher PCNA index in December than in April and August. The highest PCNA index is observed in fetal esophagi and is likely related to massive mitoses that occur during embryonic development.

Ultrastructural analysis shows that the amount and distribution of intermediate filaments in the esophagus epithelium is typical for the process of intensive keratinization, which reflects beaver’s food preferences. Intermediate filaments are involved in defense against strong mechanical stress from heterogeneous nutrition types, microorganisms, and toxins during food passage [16,34,35,36,37,38]. Special attention should be paid to the presence of variable dense bodies in the granular and keratinized layers. We distinguished two types of bodies in the granular cell layer: non-lamellar and lamellar bodies. The non-lamellar bodies are covered with a membrane and filled with electron-dense granular content and lucent clefts. In contrast, lamellar bodies are also covered with membrane, but contain electron-dense discs oriented perpendicularly or longitudinally to the long axis of those bodies. An interesting phenomenon is the rearrangement of lamellar bodies in the cytoplasm, while granular cells reach the upper epithelial layers on the border with the keratinized layer. The lamellar bodies migrate through the cell membrane to the extracellular space and form lamellar deposits. Moreover, granular bodies are arranged in irregular areas near the cell membrane, similar to lamellar bodies also found in the intercellular space. The majority of current knowledge on dense bodies in epithelial cells derives from the study performed on the epidermis. Epithelial lamellar bodies play a major role in skin barrier formation [39,40,41,42,43,44], but are also responsible for proper desquamation of epidermis and skin health [45,46,47]. Secretion of granule content to the intracellular space in the epidermis protects the keratinized layer and lower layers of cells from physical and chemical factors. Moreover, cell bodies may affect the flow between keratinized cells, regulating the permeability of the epithelial barrier [48,49,50,51,52]. In the present study, we also demonstrate that lipid droplets are present in keratinized layer. Although we are not able to identify their biochemical composition, it is possible that these droplets contain sphingolipids and glucosylceramides, as it was shown in other squamous epithelia in previous studies [29,51].

The muscularis mucosa is moderately developed and shows the organization typical for a majority of mammalian species [27,53,54,55,56]. Although the thickness and the density of muscle bundles increase in a craniocaudal direction, the muscularis mucosa does not form a continuous layer even at the entrance of the esophagus to the stomach. 

The submucosa shows a typical structure, however, no esophageal glands, which are important for food translocation and mucosa protection, are observed in any region of the beavers’ esophagi. The presence and localization of glands differ distinctly between species. Mucus glands are abundant in the proximal part of the esophagus in humans and Japanese macaque [5,6]. In some carnivores, such as dogs, racoons, Japanese martens, and masked palm civets, glands are present along the entire length of the organ [5]. In turn, mucus glands are poorly developed in cats, rabbits, and rats [5]. Similar to our results, submucosal glands were not observed in guinea pigs [3]. Mucus glands play an important role in maintaining the mucinous pre-epithelial barrier of the squamous epithelium [6,57]. Taking together, our results suggest that keratinized layer of epithelium is the main protective barrier in the European beaver esophagus.

The muscularis externa in esophagi of adult beavers is formed by skeletal muscle tissue. Similarly, in other rodents, such as mice, rats, guinea pigs, and hamsters, the muscularis externa is also composed of skeletal muscle fibres [3,5,58,59]. In contrast, human and cat esophagi have both skeletal and smooth muscle components located zonally: striated muscle fibres in the upper part, smooth muscle cells in the lower part, and both types of muscle tissue in the middle part of the organ [5,6,58,59]. In ruminants and dogs, muscularis externa is composed largely of skeletal muscle tissue [5]. Structural interspecies variations in esophageal walls could be related to animal feeding patterns, but the evolutionary significance of these differences remains unknown [5,60]. Interestingly, the muscularis externa in the esophagi of fetuses of the European beaver at the end of pregnancy comprises both striated and smooth muscle tissue. Our data agree with other studies showing that the developing rodent esophagi is initially composed of smooth muscle tissue that is later gradually replaced by striated muscle in a craniocaudal progression [58,61].

## 5. Conclusions

The present study shows that the structure of esophagus epithelium in the European beaver is similar to that observed in the skin epidermis. The process of epithelial keratinization occurs in both the adult and fetal animals, suggesting that it is genetically programmed. The thickness of the keratinized layer is season-dependent, and in winter it is 2–3-fold greater than that in summer. Interestingly, the epithelial cell layer thickness remains unchanged regardless of the season. Higher index of proliferation is observed in esophagi collected in winter compared to those collected in spring and summer. Ultrastructural studies reveal that epithelial cells contain lamellar and non-lamellar bodies, which gradually change their morphology and location while reaching the upper epithelial layers. It would be interesting in future studies to investigate cell differentiation using immunohistochemistry for different cytokeratins and for other molecules such as filaggrin, the expression of the genes responsible for keratinization of the epithelium in esophagi of the European beaver, and determine biochemical composition of lipids that are present in keratinized layer. The esophagus of the European beaver does not contain mucous glands, so the keratinized layer is a sole protective barrier against the injury by food components and gastric juice.

## Data Availability

The data presented in this study are available on request from the corresponding author.

## References

[1] Jamdar M.N., Ema A.N. (1982). The submucosal glands and the orientation of the musculature in the oesophagus of the camel. J. Anat..

[2] Windoffer R., Beil M., Magin T.M., Leube R.E. (2011). Cytoskeleton in motion: The dynamics of keratin intermediate filaments in epithelia. J. Cell. Biol..

[3] Berghes C., Tanase P., Parvu M., Dinu C., Cuca D. (2011). Contributions to the study of the esophagus and stomach morphology in guinea pig. Sci. Papers Anim. Sci. Biotech..

[4] Meyer W., Schoennagel B., Kacza J., Busche R., Hornickel I.N., Hewicker-Trautwein M., Schnapper A. (2014). Keratinization of the esophageal epithelium of domesticated mammals. Acta Histochem..

[5] Shiina T., Shimizu Y., Izumi N., Suzuki Y., Asano M., Atoji Y., Nikami H., Takewaki T. (2005). A comperative histological study of the distribution of striated and smooth muscles and glands in the esophagus of wild birds and mammals. J. Vet. Med. Sci..

[6] Zhang X., Patil D., Odze R.D., Zhao L., Lisovsky M., Guindi M., Riddel R., Bellizzi A., Yantiss R.K., Nalbantoglu I. (2018). The microscopic anatomy of the esophagus including the individual layers, specialized tissues, and unique components and their responses to injury. Ann. N.Y. Acad. Sci..

[7] Doucet C.M., Fryxell J.M. (1993). The effect of nutritional quality on forage preference by beavers. OIKOS.

[8] Nolet B.A., Rosell F. (1998). Comeback of the beaver Castor fiber: An overview of old and new conservation problems. Biol. Conserv..

[9] Haarberg O., Rosell F. (2006). Selective foraging on woody plant species by the Eurasian beaver (Castor fiber) in Telemark, Norway. J. Zool..

[10] Ziółkowska N., Lewczuk B., Petryński W., Palkowska K., Prusik M., Targońska K., Giżejewski Z., Przybylska-Gornowicz B. (2014). Light and electron microscopy of the European beaver (Castor fiber) stomach reveal unique morphological features with possible general biological significance. PLoS ONE.

[11] Squier C.A., Kremer M.J. (2001). Biology of oral mucosa and esophagus. J. Natl. Cancer Inst. Monogr..

[12] Pratama R., Schneider D., Böer T., Daniel R. (2019). First Insights Into Bacterial Gastrointestinal Tract Communities of the Eurasian Beaver (Castor fiber). Front. Microbiol..

[13] Alibardi L., Maderson P.F.A. (2003). Observations on the histochemistry and ultrastructure of the epidermis of the tuatara, Sphenodon punctatus (Sphenodontida, Lepidosauria, Reptilia): A contribution to an understanding of the Lepidosaurian epidermal generation and the evolutionary origin of the squamate shedding complex. J. Morphol..

[14] Krmpotic C.M., Carlini A.A., Galliari F.C., Favaronc P., Miglinoc M.A., Scarano A.C., Barbeitoa C.G. (2014). Ontogenetic variation in the stratum granulosum of the epidermis of Chaetophractus vellerosus (Xenarthra, Dasypodidae) in relation to the development of cornified scales. Zoology.

[15] Parakkal P.F. (1967). An electron microscopic study of esophageal epithelium in the newborn and adult mouse. Am. J. Anat..

[16] Marques-Pererira J.P., Leblond C.P. (1965). Mitosis and differentiation in the stratified squamous epithelium of the rat esophagus. Am. J. Anat..

[17] Borghesi J., Mario L.C., Carvalho R.C., Rodrigues M.N., Favaron P.O., Miglino M.A. (2015). Morphology of the digestive apparatus in Oligoryzomys nigripes (Rodentia, Sigmodontinae). Open J. Anim. Sci..

[18] Carrascal Velásquez J.C., Ortiz Bedoya S.A., Petro Hernández V.G. (2016). Microscopic characterization of esophageal regions of a group of Capybara (Hydrochoerus hydrochaeris) free in Brazil. Rev. CES Med. Zootec..

[19] Philipsen H.P., Fejerskov O. (1973). Normal histology and the effect of acute mechanical stress on the esophagus epithelium in the guinea pig. Acta Odontol. Scand..

[20] Chu E.W., Malmgren R.A. (1965). An inhibitory effect of vitamin A on the induction of tumors of forestomach and cervix in the Syrian hamster by carcinogenic polycyclic hydrocarbons. AACR.

[21] Alogninouwa T., Agba K.C., Agossou E., Kpodekon M. (1996). Anatomical, histological and functional specificities of the digestive tract in the male grasscutter (Thryonomys swinderianus, Temminck 1827). Anat. Histol. Embryol..

[22] Garcia G.W., Baptiste Q.S., Adogwa A.O., Kakuni M., Arishima K., Makita T. (2000). The digestive system of the agouti (Dasyprocta leporina)-gross anatomy and histology. Japanese J. Zoo Wildl. Med..

[23] Goetsch E. (1910). The structure of mammalian oesophagus. Am. J. Anat..

[24] Islam M.S., Awal M.A., Quasem M., Asaduzzaman M., Das S.K. (2008). Morphology of esophagus of Black Bengal goat. Bangl. J. Vet. Med..

[25] Islam M.S., Quasem M.A., Awal M.A., Das S.K. (2005). Histology of esophagus of Black Bengal goat. Bangl. J. Vet. Med..

[26] Kumar P., Mahesh R., Kumar P. (2009). Histological architecture of esophagus of goat (Capra hircus). Haryana Vet..

[27] Sokołowska J., Urbańska K., Matusiak J., Wiśniewski J. (2021). New aspects of the esophageal histology of the domestic goat (Capra hircus) and European roe deer (Capreolus capreolus). Vet. Med. Sci..

[28] Hameed B.K., Ebraheem A.H., Hussein F.A. (2018). Histological structure of the cervical segment oesophagus in goats and sheep (Comparison study). Tikrit J. Pure Sci..

[29] Gupta S.K., Sharma D.N. (1991). Regional histology of the oesophagus of buffalo calves. Indian J. Anim. Sci..

[30] Tseng S.C., Hatchell D., Tierney N., Huang A.J., Sun T.T. (1984). Expression of specific keratin markers by rabbit corneal, conjunctival, and esophageal epithelia during vitamin A deficiency. J. Cell. Biol..

[31] Rao R.S., Patil S., Ganavi B.S. (2014). Oral cytokeratins in health and disease. J. Contemp. Dent. Pract..

[32] Raymond C., Anne V., Millane G. (1991). Development of esophageal epithelium in the fetal and neonatal mouse. Anat. Rec..

[33] Yu W.Y., Slack J.M.W., Tosh D. (2005). Conversion of columnar to stratified squamous epithelium in the developing mouse oesophagus. Dev. Biol..

[34] Meyer W., Kacza J., Schnapper A., Verspohl J., Hornickel I.N., Seeger J. (2010). A first report on the microbial colonisation of the equine oesophagus. Ann. Anat..

[35] Meyer W., Kacza J., Hornickel I., Schoennagel B. (2013). Immunolocalization of succinate dehydrogenase in the esophagus epithelium of domesticated mammals. Eur. J. Histochem..

[36] Schweizer J., Bowden P.E., Coulombe P.A., Langbein L., Lane E.B., Magin T.M., Maltais L., Omary M.B., Parry D.A.D., Rogers M.A. (2006). New consensus nomenclature for mammalian keratins. J. Cell. Biol..

[37] Moll R., Divo M., Langbein L. (2008). The human keratins: Biology and pathology. Histochem. Cell. Biol..

[38] Bragulla H.H., Homberger D.G. (2009). Structure and functions of keratin proteins in simple, stratified, keratinized and cornified epithelia. J. Anat..

[39] Grayson S., Johnson-Winegar A.D., Elias P.M. (1983). Isolation of lamellar bodies from neonatal mouse epidermis by selective sequential filtration. Science.

[40] Freinkel R.K., Traczyk T.N. (1985). Lipid composition and acid hydrolase content of lamellar granules of fetal rat epidermis. J. Investig. Dermatol..

[41] Rassner U.A., Crumrine D.A., Nau P., Elias P.M. (1997). Microwave incubation improves lipolytic enzyme preservation for ultrastructural cytochemistry. Histochem. J..

[42] Ishida-Yamamoto A., Deraison C., Bonnart C., Bitoun E., Robinson R., O'Brien T.J., Wakamatsu K., Ohtsubo S., Takahashi H., Hashimoto Y. (2005). LEKTI is localized in lamellar granules separated from KLK5 and KLK7, and is secreted in the extracellular spaces of the superficial stratum granulosum. J. Investig. Dermatol..

[43] Ishida-Yamamoto A., Simon M., Kishibe M., Miyauchi Y., Takahashi H., Yoshida S., O’Brien T.J., Serre G., Iizuka H. (2004). Epidermal lamellar granules transport different cargoes as distinct aggregates. J. Investig. Dermatol..

[44] Elias P.M., Feingold K.R. (2005). Skin Barrier.

[45] Dale B.A., Holbrook K.A., Fleckman P., Kimball J.R., Brumbaugh S., Sybert V.P. (1990). Heterogeneity in harlequin ichthyosis an inborn error of epidermal keratinization: Variable morphology and structural protein expression and a defect in lamellar granules. J. Investig. Dermatol..

[46] Milner M.E., O’Guin W.M., Holbrook K.A., Dale B.A. (1992). Abnormal lamellar granules in harlequin ichthyosis. J. Investig. Dermatol..

[47] Stone S.J., Myers H.M., Watkins S.M., Brown B.E., Feingold K.R., Elias P.M., Farese R.V. (2004). Lipopenia and skin barrier abnormalities in DGAT2-deficient mice. J. Biol. Chem..

[48] Matoltsy A.G., Parakkal P.F. (1965). Membrane-coating granules of keratinizing epithelia. J. Cell. Biol..

[49] Akiyama M., Sawamura D., Nomura Y., Sugawara M., Shimizu H. (2003). Truncation of CGI-58 protein causes malformation of lamellar granules resulting in ichthyosis in Dorfman-Chanarin syndrome. J. Investig. Dermatol..

[50] Demerjian M., Crumrine D.A., Milstone L.M., Williams M.L., Elias P.M. (2006). Barrier dysfunction and pathogenesis of neutral lipid storage disease with ichthyosis (Chanarin-Dorfman syndrome). J. Investig. Dermatol..

[51] Houben E., Hachem J.P., De Paepe K., Rogiers V. (2008). Epidermal ceramidase activity regulates epidermal desquamation via stratum corneum acidification. Skin Pharmacol. Physiol..

[52] Menon G.K., Orso E., Aslanidis C., Crumrine D., Schmitz G., Elias P.M. (2014). Ultrastructure of skin from Refsum disease with emphasis on epidermal lamellar bodies and stratum corneum barrier lipid organization. Arch. Dermatol. Res..

[53] DeNardi F.G., Riddell R.H. (1991). The normal esophagus. Am. J. Surg. Pathol..

[54] Nagai K., Noguchi T., Hashimoto T., Uchida Y., Shimada T. (2003). The organization of the lamina muscularis mucosae in the human esophagus. Arch. Histol. Cytol..

[55] Christensen J., Rick G.A., Soll D.J. (1987). Intramural nerves and interstitial cells revealed by the Champy Maillet stain in the opossum esophagus. J. Auton. Nerv. Syst..

[56] Uchida K., Kamikawa Y. (2007). Muscularis mucosae—The forgotten sibling. J. Smooth Muscle Res..

[57] Sarosiek J. (2016). Does the healing of the esophageal mucosa improve the function of the esophageal submucosal and salivary glands?. Ann. N. Y. Acad. Sci..

[58] Su P.H., Wang T.C., Wong Z.R., Huang B.M., Yang H.M. (2011). The expression of nestin delineates skeletal muscle differentiation in the developing rat esophagus. J. Anat..

[59] Fullington N.M., Potanos K.M., Cauley R.P., Purcell P., Zurakowski D., Fishman S.J., Vakili K., Kim H.B. (2016). Strain induced esophageal growth in a novel rodent model. J. Pediatr. Surg..

[60] Krauss R.S., Chihara D., Romer A.I. (2016). Embracing change: Striated-for-smooth muscle replacement in esophagus development. Skelet. Muscle.

[61] Wörl J., Neuhuber W.L. (2005). Ultrastructural analysis of the smooth-to-striated transition zone in the developing mouse esophagus: Emphasis on apoptosis of smooth and origin and differentiation of striated muscle cells. Dev. Dyn..

